# VMAT‐DAT‐Dopamine Regulatory System Involved in the Protective Effect of 3‐n‐Butylphthalide Against Ischemic Stroke

**DOI:** 10.1002/cns.71094

**Published:** 2026-08-18

**Authors:** Xiao‐Ting Zhou, Qin Liu, Ming Chen, Ruo‐Xi Zhang, Yi‐Ling Su, Ding‐Feng Su, Ai‐Jun Liu

**Affiliations:** ^1^ Department of Pharmacy Research, Yueyang Hospital of Integrated Traditional Chinese and Western Medicine Shanghai University of Traditional Chinese Medicine Shanghai China; ^2^ School of Pharmacy Shandong University of Traditional Chinese Medicine Jinan China; ^3^ MOE Frontier Center for Brain Science, Institutes of Brain Science, State Key Laboratory of Medical Neurobiology Fudan University Shanghai China; ^4^ Ningbo New Drug Evaluation Center Ningbo China; ^5^ Department of Pharmacology Second Military Medical University Shanghai China

**Keywords:** 3‐n‐butylphthalide (NBP), adenosine A1 receptors (A_1_Rs), D_1_ receptor (D_1_R), dopamine, dopamine transporter (DAT), ischemic stroke, vesicular monoamine transporter (VMAT)

## Abstract

**Aims:**

A stable uptake‐release cycle system of dopamine is dependent upon two transporters, dopamine transporter (DAT) and vesicular monoamine transporter (VMAT). Whether the regulation system of dopamine is involved in the neuroprotection mediated by 3‐n‐butylphthalide (NBP) against ischemic stroke injury is unclear.

**Methods:**

Middle cerebral artery occlusion (MCAO) was subjected to evaluate the protection of NBP. In vivo microdialysis measurements of neurotransmitters were recorded in the ischemic penumbral area. Brain slices were prepared in MCAO mice to record synaptic function, including miniature excitatory postsynaptic currents (mEPSCs) and miniature inhibitory postsynaptic currents (mIPSCs).

**Results:**

NBP decreased the infarct size with a dose‐dependent manner, and significantly reduced the level of glutamate, while markedly increasing dopamine release. The expressions of DAT and VMAT were significantly decreased by MCAO, which were reversely enhanced by NBP. Pretreatment with VMAT inhibitor, reserpine, abolished the dopamine increase and eliminated the neuroprotection of NBP. The balance between excitatory and inhibitory synaptic transmission is disturbed by ischemic injury and reversed by NBP. Reserpine eliminated the effect of NBP against the disturbance of mEPSCs and mIPSCs. Inhibition of dopamine‐1 receptor (D_1_R) or adenosine A_1_ receptors (A_1_R: downstream of D_1_R) both eliminated the regulation of NBP on the synaptic function and abolished the neuroprotection of NBP.

**Conclusion:**

The neuroprotective effect of NBP against cerebral ischemic injury may involve the upregulation of the VMAT‐DAT‐dopamine cycle, which appears to contribute to the amelioration of synaptic dysfunction. The activation of D_1_R and its downstream adenosine A_1_Rs is involved in the neuroprotection mechanism.

## Graphical abstract

Proposed mechanism underlying NBP protection against ischemic stroke. NBP exerts neuroprotection against cerebral ischemic injury partially via upregulating the VMAT‐DAT‐dopamine cycle to improve synaptic dysfunction. Activation of D_1_R and adenosine A_1_Rs participates in the relevant downstream signalling cascades.

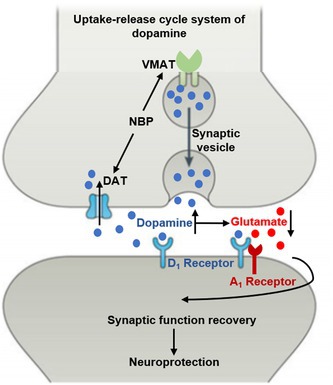

**Abbreviations**
A_1_Rs: adenosine A1 Receptors
ANOVA: analysis of variance
CBF: cerebral blood flow
CMC‐Na: Carboxymethylcellulose Sodium
D_1_R: Dopamine‐1 receptor
DAT: dopamine transporter
E/I: excitatory and inhibitory
GABA: γ‐aminobutyric acid
i.g.: intragastric
MCA: middle cerebral artery
MCAO: middle cerebral artery occlusion
mEPSCs: miniature excitatory postsynaptic currents
mIPSCs: miniature inhibitory postsynaptic currents
NBP: 3‐n‐butylphthalide
SNpc: substantia nigra pars compacta
TH: tyrosine hydroxylase
TTC: 2, 3, 5‐triphenyltetrazolium chloride
VMAT: vesicular monoamine transporter
VTA: ventral tegmental area

## 1 Introduction

Each year in the United States, ≈795,000 people experience a new or recurrent stroke [1]. Globally, stroke is the second leading cause of death (about 7 million) and the third leading cause of death and disability combined [2, 3, 4]. Ischemic stroke constituted about 65.3% of incident strokes, with the highest proportion of ischemic stroke in high income countries [2, 3, 4]. Common disabilities after stroke include motor disorders and facial central paralysis, and have a great impact on the quality of life for patients. Epidemiology study shows that the number of people who suffer a stroke, die from, or live with a disability after a stroke has risen substantially worldwide between 1990 and 2021. The estimated global cost of stroke is over US$890 billion per year, and is projected to almost double by 2050 [3].

The pathogenesis of ischemic stroke has not been fully understood, and the treatments for it are limited. To date, the only effective treatment for acute ischemic stroke is thrombolytic therapy with tissue plasminogen activator (tPA) [5]. However, due to the narrow treatment time window (within 4.5 h) and the side effects, the use of tPA is greatly restricted. It's critically important to understand the biological processes and pathogenesis of ischemic injury of stroke and develop new therapies to improve clinical outcomes.

Dopamine is an important catecholaminergic neurotransmitter and has a crucial role in a variety of behavioral processes including motor control, cognition, and emotional processing [6, 7]. Plasma membrane reuptake transporter (DAT) and the vesicular monoamine transporter (VMAT) play an important role in the regulation of transmitter release and the signaling dynamics of dopamine [8, 9]. These form a stable uptake‐release cycle system of dopamine. Recently, we reported that high level of dopamine might be an important protective factor against ischemic stroke. Targeting the DAT‐dopamine system could be explored as a new strategy for ischemic stroke [10].

3‐n‐butylphthalide (NBP) was originally extracted from the seeds of *Apium graveolens* Linn. (celery). Studies have demonstrated that NBT has significant neuroprotective effects, and has been approved as an anti‐ischemic drug in China Since 2002 [11]. Many researches involving stroke animal models have also concluded that NBP, through multiple mechanisms, significantly reduces the cerebral infarct size and oxidative damage, inhibits platelet aggregation, improves blood flow and mitochondrial functions, possesses antithrombotic and anti‐inflammatory effects, and reduces neural apoptosis [12, 13, 14, 15, 16].

However, there are few studies on the molecular targets of NBP. The targets identification is not only the key to further revealing the neuroprotective mechanism, but also important clues to develop new therapeutic indications. In this study, we investigate the potential targets of NBT by detecting neurotransmitters and conclude that the regulation of dopamine, involving DAT and VMAT might be an important molecular mechanism of NBP against ischemic stroke injury.

## 2 Methods

### 2.1 Animals

ICR mice were purchased from Sino‐British SIPPR/BK Lab Animal Ltd. (Shanghai, China). The animals were housed with controlled temperature (25°C ±) and lighting (12 h light and 12 h dark), and with free access to tap water and food. All the animals received humane care in compliance with Yueyang Hospital of Integrated Traditional Chinese and Western Medicine, Shanghai University of Traditional Chinese Medicine for health and care of experimental animals. All experiments were approved by Yueyang Hospital.

### 2.2 Reagents

NBP were obtained from CSPC NBP Pharmaceutical Co. Ltd. (Shijiazhuang, Hebei Province, China). Antibodies information: VMAT1 (HUABIO, Hangzhou, Zhejiang Province, China; Catalog# R1307‐7), VMAT2 (Bioss, Beijing, China; Catalog# bs‐9565R), DAT (ABclonal, Wuhan, China; A25875) and DAT (phospho T53, P‐DAT) (Bioss, Beijing, China; Catalog# bs‐1408R); secondary antibody IgG‐HRP (Abmart, Shanghai, China; Catalog# ABMARTM21001S). D_1_R antagonist SCH‐23390, adenosine A_1_ Receptors (A_1_Rs) antagonist PSB36 were purchased from Aladdin (Shanghai, China). Dopamine and glutamate were purchased from Sigma‐Aldrich (Shanghai) Trading Co. Ltd. (Shanghai, China). Anti‐Tyrosine Hydroxylase antibody [RM1085] was purchased from Abcam (Cambridge, UK).

### 2.3 Middle Cerebral Artery Occlusion (MCAO) and Evaluation of Neurological Deficit Scores and Infarct Size

Male mice were subjected to transient ischemia via MCAO as described previously [10]. Animals were anesthetized with 3.0% isoflurane and inhaled 1.5% isoflurane in air spontaneously by using a mask (Core body temperature was maintained at 37°C). The left MCAO surgery was subjected in mice. Nylon filaments were used (for mice 20 ± 2 g, length 33 mm, diameter 0.14 ± 0.01 mm and top diameter 0.20 ± 0.01 mm, top length 6 mm) (Beijing cinontech Co. Ltd., Beijing China). A nylon filament was inserted into the external carotid artery and gently advanced through the internal carotid artery until its tip occluded the origin of the middle cerebral artery (MCA), and external carotid artery was occluded permanently. The change of cerebral blood flow (CBF) supplied was confirmed by a laser Doppler transducer (MNP110XP, ADInstruments, Australia) connected to a laser Doppler computerized main unit (ML191, ADInstruments, Australia). Animals that did not show a CBF reduction of at least 70% were excluded from the experimental group, as were animals that died after ischemia induction. One hour after MCAO, the occluding filament was withdrawn to allow reperfusion.

Sham operation animals underwent the same surgical procedure as the MCAO group, including anesthesia, skin incision, and exposure of the common carotid artery, but without the insertion of the filament.

Seventy‐two hours after operation, neurological deficit scores of 5 points were recorded accordingly and made some revision [10, 17]. Briefly, animals without observable deficit were scored as 0, flexion of the contralateral torso and forearm on lifting the animal by the tail as 1, circling to the contralateral side but normal posture at rest as 2, leaning to the contralateral side as 3, no spontaneous motor activity as 4, and death as 5.

Then animals were anesthetized and killed by high dose of sodium pentobarbital (10%, 2.0 mL/kg, intraperitoneal injection). The brain tissue was collected and frozen at −20°C for 30 min before being cut into 6 coronal sections and incubated in 2% 2,3,5‐triphenyltetrazolium chloride (TTC) [10, 17]. The possible interference of brain edema in assessing the infarct size was corrected with a standard method of subtracting the volume of the nonischemic ipsilateral hemisphere from that of the contralateral hemisphere. The infarct size was quantified with ImageJ software (NIH, Bethesda, MD, USA).

### 2.4 NBP Administration

Mice were administered vehicle control (0.5% Carboxymethylcellulose Sodium (CMC‐Na)) or different doses of NBP (equal volume, intragastric administration (i.g.)) immediately after MCAO for the first time, then the administration was performed twice at 24 and 48 h, respectively.

### 2.5 Microdialysis

The ischemic penumbral area is the major target for neuroprotective drugs; the hippocampus in the ischemic penumbral area was selected to monitor metabolic parameters by microdialysis.

In vivo microdialysis operation and measurements of extracellular neurotransmitter levels (dopamine, glutamate) in freely moving mice were performed as described previously. The mice were placed in a stereotaxic micromanipulator (ASI Instruments, Warren, MI, USA). The guide cannula (for later microdialysis probe insertion) was implanted into the top of hippocampus (anteroposterior (AP) −2.0 mm from bregma, mediolateral (ML) −2.0 mm, dorsoventral (DV) −2.3 mm) and fixed with acrylic bone cement (The site is in the penumbra area from CA1 of hippocampus after MCAO). After 72 h, a 1 mm‐length microdialysis probe (CMA/7 microdialysis AB, Holliston, MA, USA) with an outer diameter of 0.24 mm and a molecular weight cut‐off of 6 kDa was slowly inserted into the guide cannula.

Microdialysis was used to collect basal levels (perfusion flow rate: 1 μL/min). One day later, MCAO was performed as described previously on the left side. Dialysis samples were collected in tubes containing 0.1 M perchloric acid at 1, 6, 24 and 72 h after MCAO. Keep the perfusate temperature at 37.0°C–38.0°C during dialysis. Dialysis samples were stored in liquid nitrogen for detection. Dialysis samples were assayed for dopamine by using high performance liquid chromatography—electrochemistry (HPLC‐EC, BAS PM‐92E/LC‐4C, USA) as described with some modifications [18]. Glutamate concentration measurement was performed by HPLC with the use of derivatization and fluorescence detection, essentially as previously described [19, 20, 21].

### 2.6 Brain Slice Preparation and Whole‐Cell Voltage‐Clamp Recording

Brain slices were prepared from mice at different stages of ischemic injury as the reference reported [10]. Miniature Excitatory Postsynaptic Currents (mEPSCs) and miniature inhibitory postsynaptic currents (mIPSCs) were recorded accordingly.

Brain slices were prepared from animals at different stages (0, 6, 24 and 72 h) after ischemic injury. The animals were anesthetized with 3.0% isoflurane and decapitated. The brains were quickly removed and immersed in ice‐cold artificial cerebrospinal fluid (ACSF). Coronal slices of 250 μm thickness were cut using a vibratome (VT1200S, Leica, Nussloch, Germany) and incubated in oxygenated ACSF for 1 h at 32°C.

Recordings were performed in penumbra area (which can be clearly defined by microscope) in CA1 of hippocampus at room temperature (24°C). For whole‐cell recording, patch electrodes were prepared from borosilicate glass (Φ 1.50 mm, *φ* 0.89 mm, Vital Sense Scientific Instruments 20132139, China) using a horizontal electrode puller (Sutter P‐97, USA). The resistance of the pipette filled with intracellular solution was 2–3 ΜΩ.

For mEPSCs recording, electrodes were filled with an intracellular solution containing (mM): K‐gluconate 133, NaCl 8, EGTA 0.6, Mg·ATP 2, Na_3_·GTP 0.3, HEPES 10. To allow verification of the identity of recorded neurons, sodium channel blocker tetrodotoxin (TTX 0.5 μM) and picrotoxin 100 μM were included in the solution. The membrane potential was clamped at −70 mV and recorded the mEPSCs for 15 min.

For miniature inhibitory postsynaptic currents (mIPSCs) recording, electrodes were filled with an intracellular solution containing (mM): KCl 130, MgCl_2_ 1.0, EGTA 5, Na_2_·ATP 5, HEPES 5. To allow verification of the identity of recorded neurons, TTX 0.5 μM and non‐NMDA receptor blocker 6,7‐dinitroquinoxaline‐2,3‐dione (DNQX, 10 μM) were included in the solution. Detection voltage and recording time were the same as above.

### 2.7 Tyrosine Hydroxylase (TH) Immunostaining

To evaluate the effects of NBP on the dopamine and noradrenergic projections, TH immunostaining was performed in the brain of MCAO mice. NBP and vehicle were administrated for 3 days after MCAO. Then the brains were perfusion‐fixed with 4% paraformaldehyde in 0.1 M phosphate buffer (pH 7.4) following 0.9% saline flush. The brains were removed and immersed in the same fixative until they were embedded in paraffin. Paraffin sections (5 μm) of the striatum and substantia nigra were used for immunohistochemistry.

### 2.8 Immunoblotting

The total protein was prepared from ischemic penumbral area from hippocampus and adjacent cerebral cortex using lysis buffer containing 50 mM Tris–HCl (pH 6.8), 2% Sodium Dodecyl Sulfate (SDS), 10% glycerol, and 100 mM dithiothreitol (DTT), supplemented with protease and phosphatase inhibitors. To ensure complete solubilization of hydrophobic membrane proteins, the tissue homogenates were vigorously sonicated on ice for 3 min (5 s pulses) before boiling. Tissue extract was boiled in 4× loading buffer, subjected to SDS‐PAGE, and transferred onto the pure nitrocellulose blotting membranes. The membranes were incubated with anti‐DAT, DAT (phospho T53, P‐DAT), VMAT1, VMAT2 antibody prior to incubation with secondary antibody. The image was captured by the ChemiDoc MP imaging system (Bio‐Rad Laboratories, Hercules, California, USA). ImageJ software (NIH) was used to analyze the images. All experiments were repeated for 3 times.

### 2.9 Statistical Analysis

The random permutations table was used to randomly assign mice. The order of experiment operation was alternated between different groups. The investigators were blinded during the procedures of operation and measurement. Results were plotted with the Software of GraphPad Prism 7.00 (GraphPad Software, San Diego, CA, USA). Data are expressed as mean ± standard deviation. Normality distribution is evaluated by Shapiro–Wilk test. Data are analyzed with 2‐tailed Student's *t*‐test (2 groups) and one‐way or two‐way analysis of variance (ANOVA) (> 2 groups) after passing normality test of variance. Otherwise, data are analyzed by using a non‐parametric equivalent. *p* < 0.05 is considered statistically significant.

## 3 Results

### 3.1 NBP Protected Against Ischemic Cerebral Injury in Mice

**FIGURE 1:**
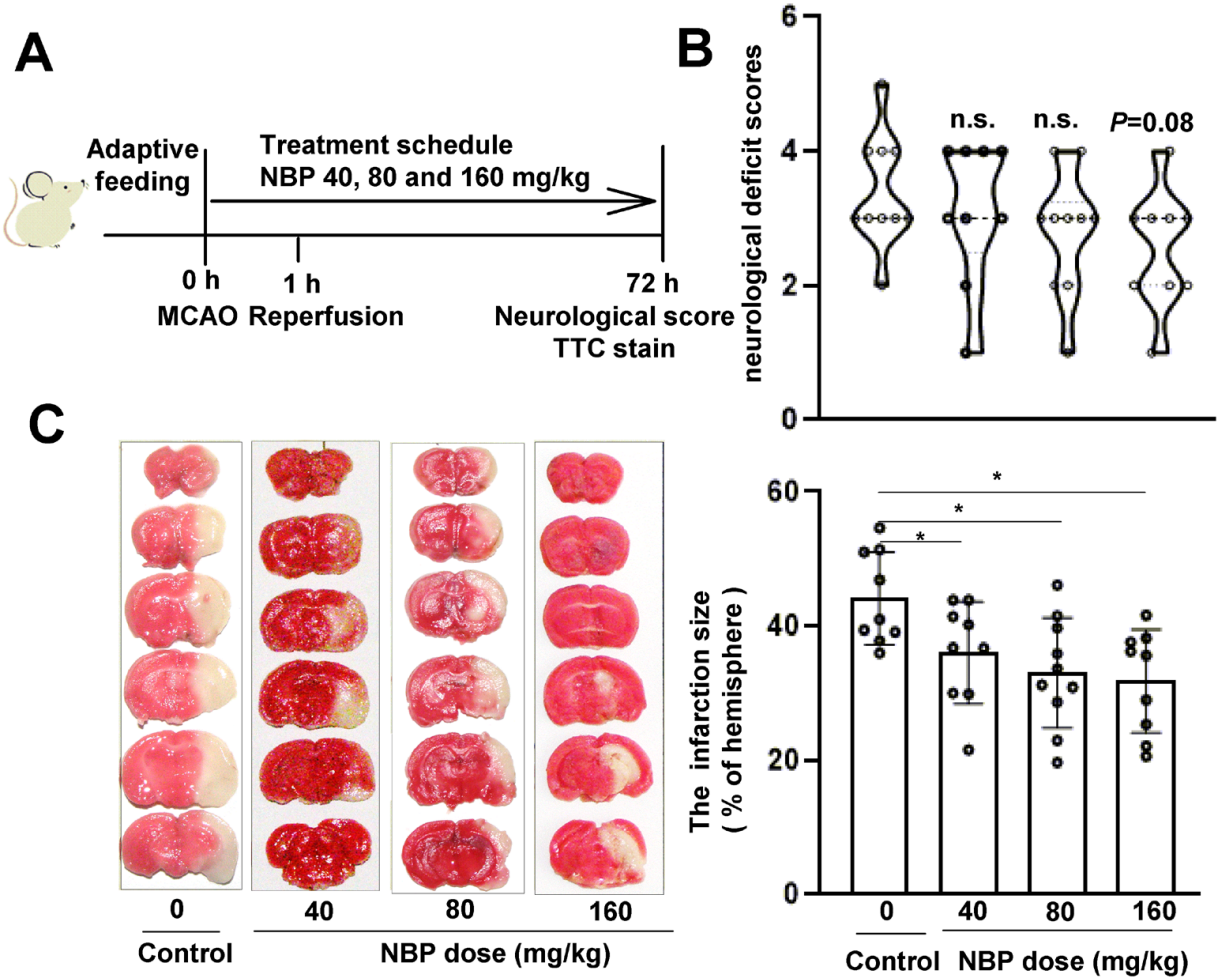
NBP reduced the infarct size and improved neurological function in mice with MCAO. (A) Time schedule and dose regimen. (B) The neurological deficit scores at 72 h after MCAO. (C) Representative 1% TTC staining images of coronal brain sections (Left) and the measurement of infarction size. Mice were administered vehicle (0.5% CMC‐Na, i.g., *n* = 10) or NBP (40, 80 and 160 mg/kg, i.g., *n* = 10) immediately after MCAO for the first time, then the administration was performed twice at 24 h and 48 h, respectively. Data are shown as mean ± SD and analyzed by ANOVA testing followed by Dunnett's testing, or non‐parametric Kruskal–Wallis test for neurological deficit scores (Dunn's post hoc test was applied). *p < 0.05 versus control group. ns, no significance.

To define the role of NBP against stroke, 40 male ICR mice (20 ± 2 g) were randomly divided into 4 groups (vehicle control and 3 NBP treatment groups) and subjected to MCAO following NBP (40, 80 and 160 mg/kg) or vehicle administration immediately for the first time; then the administration was performed twice at 24 h and 48 h, respectively (Figure 1A). TTC staining and neurological scores were used to evaluate the effects of different doses of NBP on the cerebral injury. Three animals were discarded because of death (control) or low CBF reduction (40 and 160 mg/kg group). As shown in Figure 1C and Figure S1, NBP decreased the infarct size in a dose‐dependent manner (36.0% ± 7.55% in 40 mg/kg; 33.0% ± 8.19% in 80 mg/kg; 31.8% ± 7.69% in 160 mg/kg vs. 44.1% ± 6.90% in control group, *p* < 0.05, Figure 1C). The neurological function was improved but not significantly (*p* > 0.05, Figure 1B).

### 3.2 The Acute Changes of Dopamine and Glutamate in MCAO Mice Were Regulated by NBP

**FIGURE 2:**
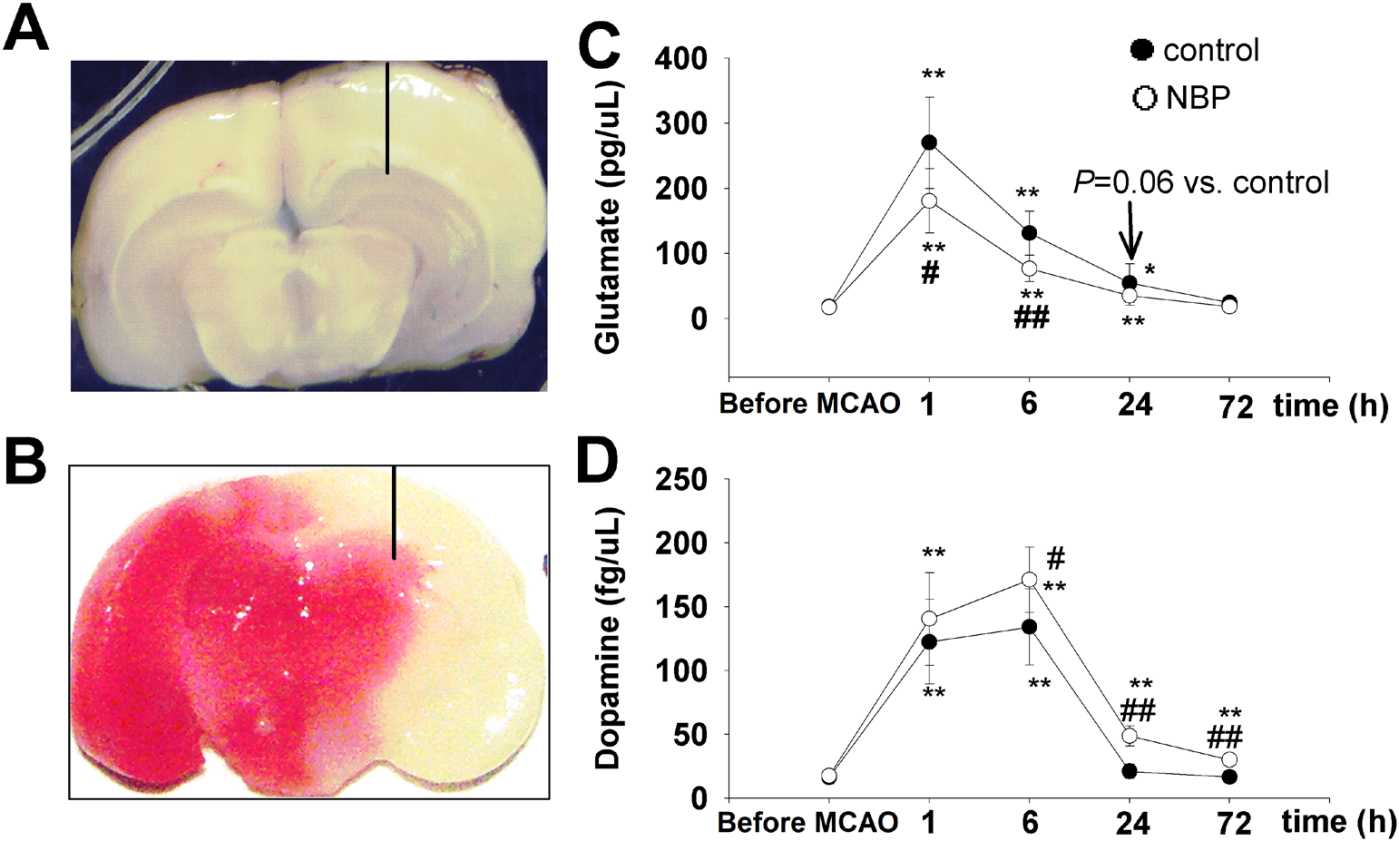
Dopamine and glutamate were increased in the ischemic penumbra of MCAO mice. In vivo microdialysis detection shows the change of extracellular concentrations of dopamine and glutamate at different time after MCAO (*n* = 6). (A, B) The probe was located in the ischemic penumbra from CA1 of hippocampus as indicated. Ischemic brain slice without staining (A) or stained with 1% TTC (B). (C, D) The change of glutamate (C) and dopamine (D) before operation and at 1, 6, 24 and 72 h after MCAO. Data are shown as mean ± SD and analyzed by two‐way ANOVA, followed by Dunnett's test. *p < 0.05, **p < 0.01 versus before MCAO (0 h). #*p* < 0.05, ##*p* < 0.01 versus control group.

To investigate the effect of NBP on the change of important extracellular neurotransmitters during ischemic injury, male ICR mice (20 g ±, *n* = 6 in each group) were randomly divided into 2 groups (vehicle control and NBP 80 mg/kg treatment groups) and subjected to MCAO. Microdialysis method was used in freely moving mice to detect the extracellular levels of dopamine and glutamate in the ischemic penumbral area from CA1 of hippocampus (Figure 2A,B).

Before MCAO, the extracellular concentration of glutamate was 18.6 ± 6.37 pg/μL. The level of glutamate was significantly increased by about 15‐fold (270 ± 69.9 pg/μL, *p* < 0.01) in the first hour after MCAO. Then the level gradually decreased and remained at higher level at 24 h by about 3‐fold (54.6 ± 21.8 pg/μL, *p* < 0.05). The concentration of glutamate recovered to 24.3 ± 5.25 pg/μL at 72 h (Figure 2C). Compared to the vehicle control group, the level of glutamate was significantly decreased in NBP group at the first hour (180 ± 17.2 vs. 270 ± 69.9 pg/μL, *p* < 0.05) and at 6 h (76.6 ± 20.4 vs. 131 ± 33.7 pg/μL, *p* < 0.01).

MCAO caused a prominent increase of dopamine in the first hour after MCAO (122 ± 33.2 vs. 16.0 ± 4.98 fg/μL [before MCAO], *p* < 0.01). Dopamine was also strongly increased at 6 h after MCAO (134 ± 30.0 fg/μL) and recovered after 24 h to baseline levels (Figure 2D). Compared to the vehicle control group, the level of dopamine was increased in the NBP treatment group at 6 h (171 ± 25.7 vs. 134 ± 30.0 fg/μL, *p <* 0.05). The concentration of dopamine in NBP group remained at higher levels at 24 h (48.6 ± 7.93 vs. 20.8 ± 5.71 fg/μL in vehicle control group, *p* < 0.01) and 72 h (29.9 ± 3.13 vs. 16.5 ± 3.40 fg/μL in vehicle control group, *p* < 0.01). All these data suggest that NBP could increase dopamine and reduce glutamate during acute cerebral ischemic injury.

### 3.3 NBP Did Not Affect the Loss of TH Immunostaining Positive Cells in MCAO Mice

To evaluate the effects of NBP on dopaminergic projections, TH immunostaining was performed on brain sections from MCAO mice. The mice were randomly assigned to three groups: sham operation group, model vehicle control group, and NBP (80 mg/kg) treatment group (*n* = 5 per group).

Representative images of TH immunostaining in the substantia nigra and ventral tegmental area (VTA) are presented in Figure S2A. In sham mice, the substantia nigra exhibited intense TH staining, with clearly visible immunopositive neuronal somata. In the ischemic hemisphere of MCAO mice, both immunopositive fibers and cell bodies in the substantia nigra were markedly reduced. However, no significant difference was observed between the model vehicle control and NBP treatment groups (Figure S2A,C). Faint TH immunostaining was noted in both the VTA and hippocampus (Figure S3), and there were no significant differences among the three groups in these regions (Figures S2B, S3). Collectively, these findings suggest that NBP does not attenuate the ischemic injury‐induced loss of dopaminergic cells in the substantia nigra.

### 3.4 The Regulation of Dopamine Transporter (DAT) and the Vesicular Monoamine Transporter (WMAT) by NBP Involved in the Increase of Dopamine

The regulation of synaptic dopamine is dependent upon proper functioning of two transporters, named the dopamine transporter (DAT, altering dopamine reuptake activity) and the vesicular monoamine transporter (VMAT, regulating dopamine release) including VMAT1 and VMAT2 [18, 19].

**FIGURE 3:**
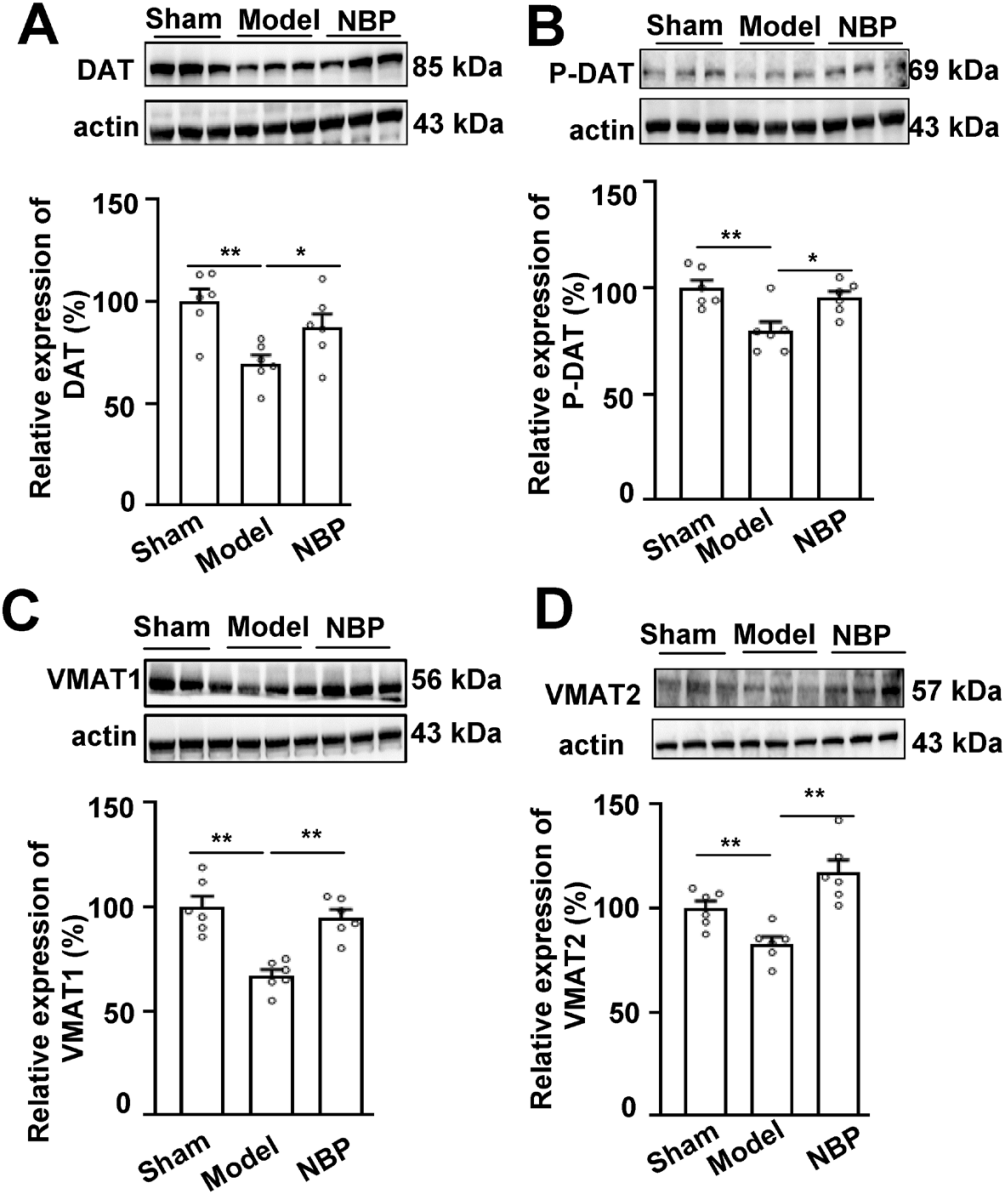
NBP increased the proteins expression related to dopamine regulation system in MCAO mice. Immunoblotting and quantification showing the expression of DAT (A), P‐DAT (B), VMAT1 (C) and VMAT2 (D) in the ischemic penumbra of mice at 24 h after MCAO. *n* = 6. mean ± SD. One way ANOVA was used, followed by Dunnett's test. *p < 0.05, **p < 0.01, ns, no significance.

To investigate whether these transporters were involved in the upregulation of dopamine by NBP, male ICR mice (20 ± 2 g) were randomly divided into 3 groups (sham, MCAO vehicle control and NBP 80 mg/kg treatment groups, *n* = 6 in each group) and subjected to MCAO. After 24 h, the ischemic penumbral area of brain from hippocampus and adjacent cerebral cortex was selected for immunoblotting determination. Compared to the sham group, the expression of DAT was significantly decreased in MCAO group (69.6% ± 10.2% vs. 100% ± 15.1% in sham group, *p* < 0.01). NBP treatment enhanced the expression of DAT by about 26% (*p* < 0.05, Figure 3A). Compared to the vehicle control group, NBP also significantly increased the expression of P‐DAT (*p* < 0.05, Figure 3B). Similar results were found in the VMAT determination. MCAO caused a significant reduction in the expression of VMAT1 (67.0% ± 7.29% vs. 100% ± 12.9% in sham group, *p* < 0.01, Figure 3C). and VMAT2 (78.5% ± 5.26% vs. 100% ± 9.08% in sham group, *p* < 0.01, Figure 3D). Compared to the MCAO vehicle control group, the expressions of VMAT1 and VMAT2 were significantly enhanced by 41.4% and 39.8%, respectively by NBP (*p* < 0.01, Figure 3C,D). All these data indicated that the NBP might alleviate the injury of synaptic function for reuptake and release of dopamine induced by MCAO.

### 3.5 The Inhibitor of VMAT by Reserpine Abolished the Protection of NBP Against Ischemic Cerebral Injury

**FIGURE 4:**
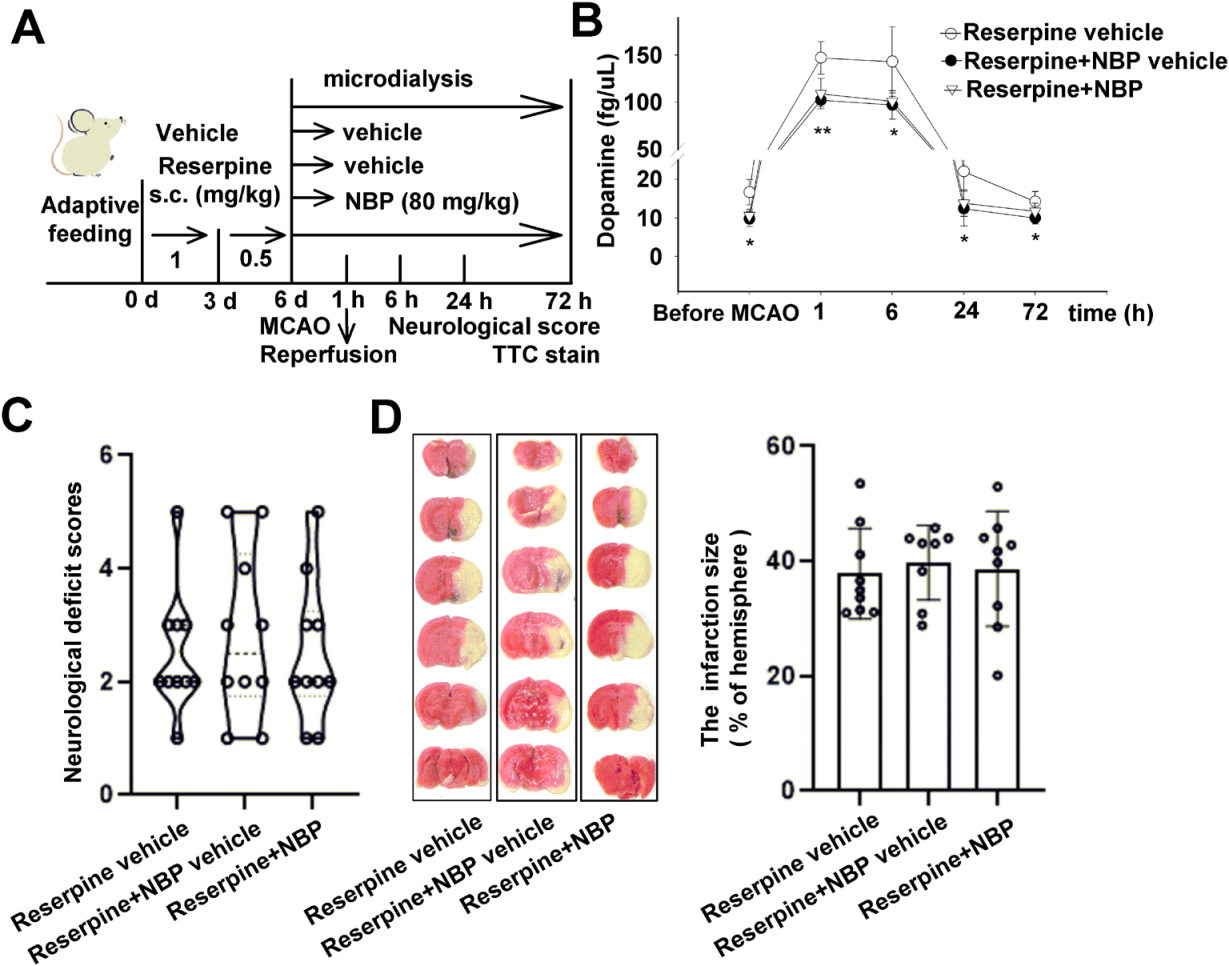
The inhibition of VMAT by reserpine abolished the protection of NBP against ischemic cerebral injury. (A) Time schedule, groups and dose regimen. (B) The change of dopamine before operation and at 1 h, 6 h, 24 h and 72 h after MCAO, *n* = 5. (C) The neurological deficit scores at 72 h after MCAO. (D) Representative 1% TTC staining images of coronal brain sections (Left) and the measurement of infarction size. Mice were administered reserpine (1 mg/kg, subcutaneous injection) or vehicle (0.5% acetic acid solution) and followed by NBP (80 mg/kg, i.g.) and vehicle (0.5% CMC‐Na), *n* = 10. Data are shown as mean ± SD and analyzed by two ways (B) or one way (D) ANOVA, followed by Dunnett's test. Non‐parametric Kruskal–Wallis test for (C), and Dunn's post hoc test was applied. *p < 0.05, **p < 0.01, versus reserpine vehicle; ns, no significance versus reserpine group in (B).

We designed experiments to investigate whether dopamine and its regulation system were involved in the protective effect of NBP. Reserpine is an indole alkaloid and can work through competitively inhibiting both VMAT isoforms [18]. In the following experiment, reserpine was used as a tool drug to reduce the level of dopamine in brain [20]. Firstly, male ICR mice (20 g ±) were randomized divided into 3 groups and administered vehicle (0.5% acetic acid solution, *n* = 15) or reserpine for 6 days (subcutaneous injection, 1 mg/kg for 3 days following 0.6 mg/kg for another 3 days, *n* = 30). Then animals were subjected to MCAO following treatment with vehicle (0.5% CMC‐Na) or NBP (80 mg/kg) (i.g., *n* = 15 in each group) (Figure 4A).

The level of dopamine in the ischemic penumbral area from CA1 of hippocampus was detected by microdialysis method before MCAO and 1, 6, 24 and 72 h after MCAO. (*n* = 5 in each group). The other 30 animals were used to evaluate the neurological function and infarct size at 72 h after MCAO. Four mice died after operation (1 in vehicle control, 2 in reserpine group, 1 in reserpine + NBP group, neurological deficit score of these mice is 5).

Before MCAO, the extracellular level of dopamine was significantly reduced by reserpine (9.75 ± 1.96 fg/μL vs. 16.7 ± 3.26 fg/μL in reserpine vehicle group *p* < 0.05, Figure 4B). In reserpine group, MCAO also caused a significant increase in dopamine at 1 h and 6 h, then returned to the baseline at 72 h. Compared to vehicle control group, dopamine was significantly reduced at 1 h (102 ± 7.27 fg/μL vs. 147 ± 17.3 fg/μL in vehicle control group *p* < 0.01), 6 h (97.1 ± 15.1 fg/μL vs. 143 ± 36.9 fg/μL *p* < 0.05), 24 h (12.4 ± 4.51 fg/μL vs. 22.0 ± 4.73 fg/μL *p* < 0.05) and 72 h (9.97 ± 1.53 fg/μL vs. 14.2 ± 2.60 fg/μL *p* < 0.05) in reserpine group. At the same time, pretreatment with reserpine almost completely abolished the dopamine increase induced by NBP. These data indicated that the NBP increasing the level of dopamine might depend on VMAT.

Compared to the vehicle control group, reserpine treatment did not affect the infarct size (39.7% ± 6.50% vs. 37.8% ± 7.90% in vehicle control group, *p* > 0.05, Figure 4D). In the NBP + reserpine treatment group, the protection of NBP was almost eliminated (38.7% ± 10.0% vs. 37.8% ± 7.90% in reserpine group, *p* > 0.05). The neurological function evaluation also got similar results. There was no significant difference between 3 groups (2.8 ± 1.5 in reserpine group, 2.5 ± 1.3 in NBP + reserpine group vs. 2.5 ± 1.1 in vehicle control group *p* > 0.05, Figure 4C). These data indicated that dopamine and VMAT might be involved in the protection of NBP against cerebral ischemic injury.

### 3.6 NBP Restored the Synaptic Function in Ischemic Penumbra of MCAO Mice

The inhibitory and excitatory synaptic transmission is very important to keep the balance of synapse function of brain. Usually, the mEPSC/mIPSC frequency is defined as a key index of this balance [21, 22]. It can be significantly disturbed by ischemic injury [10].

**FIGURE 5:**
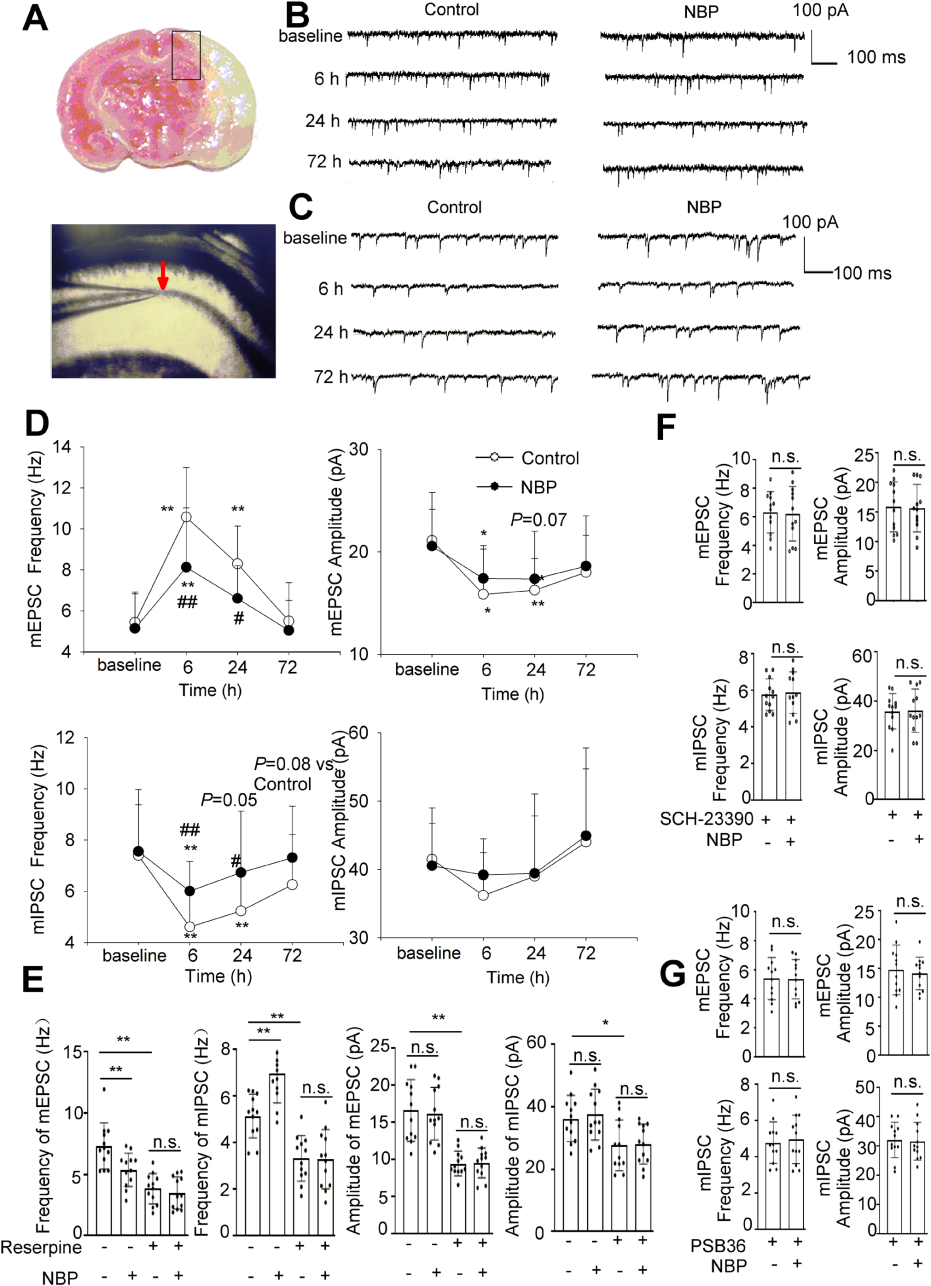
NBP restored the synaptic function in ischemic penumbra of MCAO mice. (A) Ischemic penumbra was selected for recording (black square frame in upper panel). Bright field image shows the neuron recording in penumbra (arrow, lower panel). (B–C) Representative traces of mEPSCs (B) and mIPSCs (C) in control and NBP treatment mice at different stage of ischemic injury. (D) The change of frequency (middle panel) and amplitude (right panel) at baseline and at 6, 24 and 72 h after MCAO (*n* = 12 cells/4 mice) in mice. (E) Reserpine affected the effect of NBP on the frequency and amplitude of mEPSCs and mIPSCs in MCAO animals. (F, G) D_1_R antagonist SCH‐23390 (F) or PSB36 (G) abolished the effect of NBP on the change of frequency of mEPSCs and mIPSCs. *N* = 12 cells/4 mice in (E) and (F). Data are shown as mean ± SD and analyzed by two‐way ANOVA, followed by Dunnett's test (D), one‐way ANOVA, followed by Tukey's post hoc test. (E), and Student's *t*‐test (F,G). *p < 0.05, **p < 0.01 versus before MCAO (0 h). #*p* < 0.05, ##*p* < 0.01 versus control group.

To evaluate the effects of NBP on synapse function, whole‐cell patch‐clamp recording were performed in the ischemic penumbra area of brain slices (Figure 5A). The animals were randomly assigned to divide 2 groups (the vehicle control and NBP 80 mg/kg treatment groups). NBP and vehicle were administrated for 1–3 days prior to patch‐clamp recording. The brain slices were prepared at different time points after MCAO (6, 24 and 72 h, 20 g ± 2 g, *n* = 4). For sham operation animals, NBP and vehicle were administrated before operation then brain slices were prepared immediately after the sham procedure (*n* = 4). Synaptic responses were then compared between these groups. Data from the sham‐operated groups were served as baseline. For the frequency of mEPSC, compared to the baseline (5.44 ± 1.47 Hz at baseline), the frequency of mEPSC was significantly increased at 6 h (10.6 ± 2.42 Hz *p* < 0.01) and 24 h (8.30 ± 1.84 Hz *p* < 0.01). Then the frequency recovered to baseline level at 72 h. NBP did not affect the frequency of mEPSC in sham operation animals. In MCAO mice, compared to the model control vehicle group, the frequency was significantly reduced both at 6 h (8.13 ± 2.90 Hz vs. 10.6 ± 2.90 Hz *p* < 0.01) and 24 h (6.60 ± 1.61 Hz vs. 8.30 ± 1.84 Hz *p* < 0.01) in NBP treatment mice. There's no difference between 2 groups at 72 h (Figure 5B,D).

For the amplitude of mEPSC in the vehicle control group, compared to the baseline, the amplitude value was significantly decreased at 6 h (15.9 ± 4.42 pA, *p* < 0.01) and 24 h (16.0 ± 1.84 pA, *p* < 0.01). Then the amplitude partly recovered to baseline level at 72 h (compared to 0 h, *p* > 0.05). There was no difference between vehicle control and NBP treatment groups at different time stages.

For inhibitory synaptic function, compared to the level of baseline (7.39 ± 1.98 Hz), the frequency of mIPSC was significantly decreased both at 6 h (4.61 ± 1.35 Hz, *p* < 0.01) and 24 h (5.23 ± 1.38 Hz, *p* < 0.01) after MCAO (Figure 5C,D). The frequency partly recovered at 72 h (6.26 ± 2.48 Hz, *p* > 0.01). The amplitude of mIPSC had no significant change after MCAO. Compared to the vehicle control group, the frequency mIPSC was significantly increased both at 6 h (6.01 ± 1.15 Hz vs. 4.61 ± 1.35 Hz, *p* < 0.01) and 24 h (6.73 ± 2.41 Hz vs. 5.23 ± 1.38 Hz, *p* < 0.05) in NBP treatment mice. There's no difference between 2 groups at 72 h (*p* > 0.05). These data indicate that NBP can recover the imbalance between excitatory and inhibitory synaptic transmission which is disturbed by ischemic injury.

### 3.7 Reserpine Abolished the Effect of NBP on the Restoration of Synaptic Dysfunction in MCAO Animals

Male ICR mice (20 ± 2 g) were randomized divided into 4 groups and administered vehicle or reserpine for 6 days according to the previous method. Then the animals were subjected to MCAO and treated by vehicle (0.5% CMC‐Na) or NBP (80 mg/kg) (i.g., *n* = 4 in each group). In the following experiments, only 24 h time point was examined. The brain slices were prepared 24 h after MCAO and pretreated by vehicle or NBP (10 μM).

Data are shown in Figure 5E. Reserpine pretreatment both significantly reduced the frequency of mEPSC (3.83 ± 1.24 Hz vs. 7.33 ± 1.89 Hz, *p* < 0.01) and mIPSC (3.33 ± 0.97 Hz vs. 5.16 ± 1.02 Hz, *p* < 0.01). Compared to the vehicle control group, the frequency of mEPSC was significantly decreased in NBP group (5.38 ± 1.37 Hz, *p* < 0.01). At the same time, the frequency of mIPSC was increased (6.87 ± 1.71 Hz, *p* < 0.05). NBP did not affect the amplitude of mEPSC or mIPSC. In the reserpine pretreatment groups, the frequency of mEPSC had no difference between the vehicle control and NBP groups (3.48 ± 1.31 Hz vs. 3.83 ± 1.63 Hz, *p* < 0.01). The recording of frequency of mIPSC got the similar results. Reserpine pretreatment almost eliminated the frequency increase by NBP (3.30 ± 1.27 Hz vs. 3.33 ± 0.98 Hz in reserpine alone group *p* > 0.05).

### 3.8 Dopamine 1 Receptor (D_1_R) Involved in the Effect of NBP on the Restoration of Synaptic Function

D_1_‐like receptor activation has been suggested to provide neuroprotection against ischemic injury via EPSC depression [22]. D_1_R antagonist SCH‐23390 (1 μM) was used to further evaluate the mechanism of NBP on the restoration of synaptic function. The brain slices were prepared in mice 24 h after MCAO (*n* = 8) and pretreated with SCH‐23390 (1 μM) for 30 min. Then slices were treated with NBP (10 μM, *n* = 4) and its vehicle (*n* = 4). Both the frequency of mEPSC and frequency of mIPSC had no significant difference between NBP and vehicle control groups (*n* = 12 cells, Figure 5F). For the amplitude of mEPSC and mIPSC, there was also no difference between 2 groups. D_1_R antagonist SCH‐23390 almost eliminated the regulation of NBP on synaptic function.

### 3.9 Inhibition of Adenosine A1 Receptors (A_1_Rs) Eliminated the Restoration of Synaptic Function and Protection of NBP Against Ischemic Injury

A_1_Rs can inhibit synaptic glutamate release and are defined as downstream of D_1_R activation for synaptic function and cerebral ischemic injury [23, 24]. To test whether A_1_Rs involved in the downstream mechanism of NBP, we preincubated brain slices in MCAO mice (24 h after operation) with the A_1_R antagonist PSB36 (10 μM). NBP (10 μM) failed to decrease the frequency of mEPSC or increase the frequency of mIPSC (*n* = 12 cells/4 mice, Figure 5G).

**FIGURE 6:**
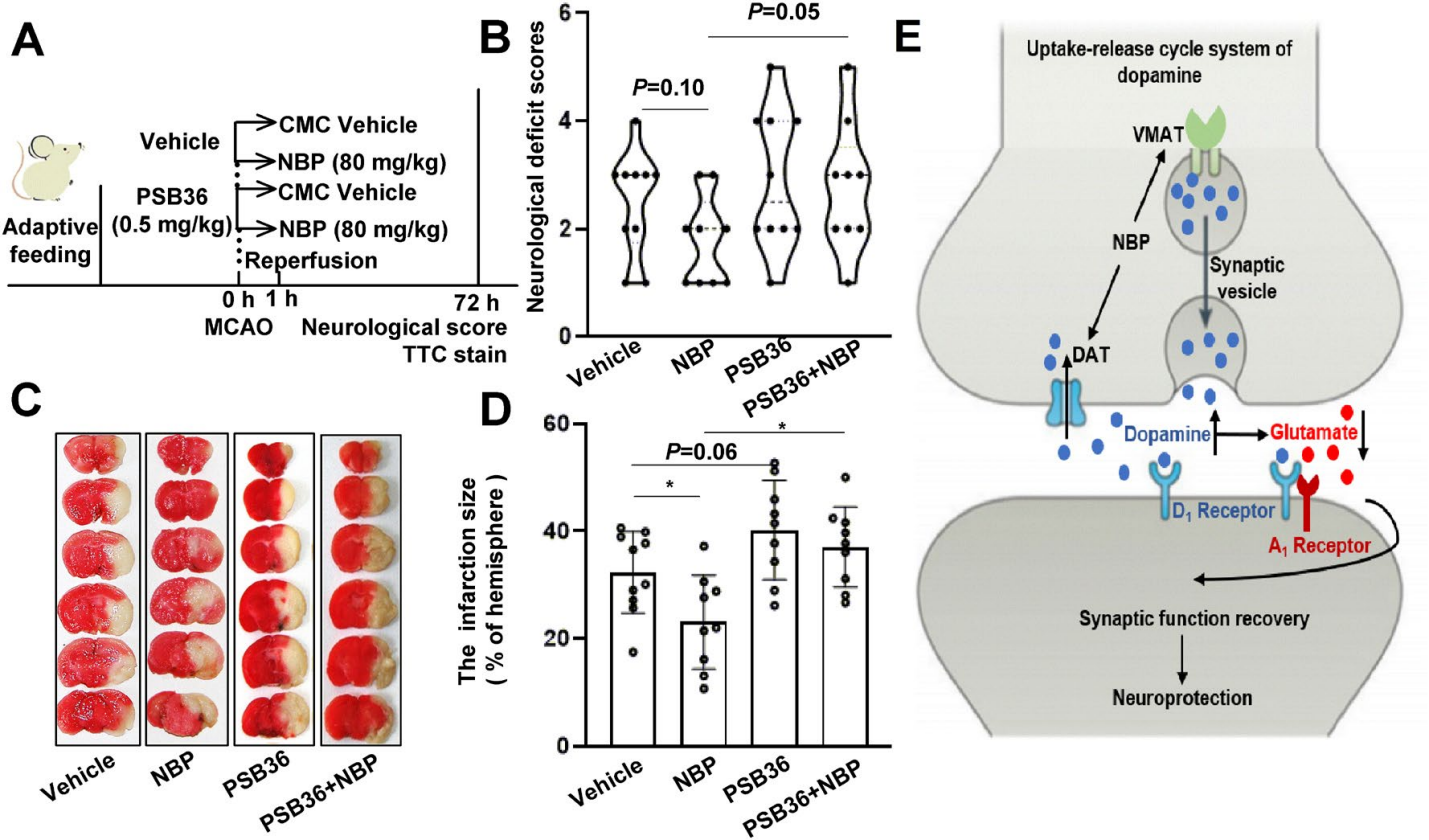
Inhibition of adenosine A_1_ Receptors (A_1_Rs) eliminated the protection of NBP against ischemic injury. (A) Time schedule and groups regimen. (B) The neurological deficit scores at 72 h after MCAO. (C) Representative 1% TTC staining images. (D) The measurement of infarction size. *N* = 9–10. Data are shown as mean ± SD and analyzed by one‐way ANOVA, followed by Tukey's post hoc test. Non‐parametric Kruskal–Wallis test for neurological scores, followed by Dunn's post hoc test. *p < 0.05, **p < 0.01. (E) Proposed mechanism underlying NBP against ischemic stroke.

Then 60 male ICR mice (20 ± 2 g) were randomly assigned to divide 4 groups and administered vehicle or PSB36 (0.5 mg/kg, intraperitoneal injection). Animals were subjected to MCAO following treatment with vehicle (0.5% CMC‐Na) or NBP (80 mg/kg) (*n* = 10 in each group) (Figure 6A). The neurological function and infarct size were evaluated at 72 h after MCAO.

Among these animals, 20 mice were used for microdialysis at 6 h after MCAO to detect the extracellular levels of glutamate in the ischemic penumbral area from CA1 of hippocampus (*n* = 5 in each group). Data are shown in Figure S4. Compared to the vehicle control group, the level of glutamate was significantly decreased in NBP group at the 6 h (79.8 ± 20.0 vs. 156 ± 30.2 pg/μL, *p* < 0.01). PSB36 pretreatment increased the level of glutamate (212 ± 48.3 pg/μL, *p* = 0.11). Pretreatment by PSB36 almost eliminated the reduction of glutamate by NBP (196 ± 48.1 vs. 212 ± 48.3 pg/μL). These data indicate that A_1_Rs may participate in NBP‐mediated reduction of glutamate.

The other animals were used to assess the cerebral infarction. PSB36 increased the infarct size (40.2% ± 9.23% vs. 32.3% ± 7.62% in vehicle control group, *p* = 0.06, Figure 6C,D). Pretreatment by PSB36 almost eliminated the reduction of infarct size by NBP (34.3% ± 6.68%). The value of neurological function was reduced by NBP (1.8 ± 0.8 vs. 2.5 ± 1.0 in vehicle control group *p* = 0.10, Figure 6B). Pretreatment by PSB36 abolished the reduction of neurological score (2.8 ± 1.2). These data indicated that A_1_Rs involved in the downstream mechanism of NBP against ischemic stroke.

## 4 Discussion

Our results demonstrate that NBP increases the dopamine level and decreases glutamate concentration during the acute ischemic injury stage through the regulation of dopamine by VMAT and DAT. Dopamine‐related D_1_R activation restores the balance of synaptic function and contributes to NBP‐induced protection against ischemic injury. Adenosine A_1_Rs might be involved in the downstream mechanism (Figure 6E).

Dopamine is an important neurotransmitter in the regulation of a variety of behavioral processes [6, 7]. Its function in ischemic stroke is controversial. Reports show that cerebral ischemic injury can cause dopamine 500‐fold increase in the neostriatum, and ischemic neuronal injury can be attenuated by dopamine depletion in the striatum of rat [25, 26].

On the other hand, the ischemia‐induced H_2_O_2_ production was not attenuated by the dopamine depletion resulting from the lesion of the substantia nigra [27]. Conversely, a selective D_1_ agonist can significantly prevent oxidation‐related necrotic cell death [28]. Indeed, the activation of dopamine receptors, D_1_R and D_2_R both have been shown neuroprotection against ischemic injury [29, 30]. Increase dopamine by levodopa can significantly recover the neurological function after MCAO in rats [31].

Our published data found that high levels of dopamine might be beneficial for ischemic stroke injury. Mutation of DAT can cause a high level of dopamine in the ischemic penumbra of the brain and significantly reduce the infarct injury induced by MCAO. The inhibition of DAT can protect against ischemic injury. Pretreated by madopar is also beneficial for ischemic stroke [10].

Combined with the above findings, we propose that the relationship between dopamine levels and cerebral ischemia may be associated with its release quantity and regional distribution in brain tissues. Different from the core ischemic area, our data showed that in the ischemic penumbral area, MCAO caused an increase of dopamine by about 8‐fold. NBP further significantly increased the dopamine level during the acute cerebral ischemic injury.

A legitimate concern is that the dialysate dopamine concentration is below the in vitro D_1_ receptor affinity. This apparent discrepancy might be explained by several reasons: microdialysis measures the low, steady‐state dopamine that has leaked out of synapses, not the high, brief bursts of dopamine that actually trigger D_1_ receptors. When dopamine uptake is working normally, the dopamine released during a nerve impulse never reaches the microdialysis probe—it is taken back up before it can diffuse that far [32]. This means microdialysis systematically underestimates the peak synaptic concentration. Cragg & Rice further showed that even after escaping the synapse, dopamine diffuses up to 10 μm to activate extrasynaptic D_1_ receptors [33]. Therefore, the dialysate value might only reflect diluted background levels, not the relevant signal for D_1_ activation.

Exhausting dopamine by VMAT inhibition almost abolished the protective effect of NBP against ischemic injury. Indeed, both VMAT and DAT are the key transporters to regulate dopamine. Dopamine is located in neurotransmitter vesicles, extracellular spaces, and extravesicular cytoplasmic compartments. Dopamine is encapsulated into vesicles via VMAT. These vesicles then merge with the membrane and release dopamine. After dopamine releasing, it can be transported back into the terminal via DAT [18]. These just form an uptake‐release cycle, and finely tuned machinery ensures to keep a stable level of dopamine and retain the normal synaptic function of neurons (Figure 6E).

There are two isoforms of VMAT: VMAT1 and VMAT2. It should be noted that VMAT1 is mainly expressed in neuroendocrine cells while VMAT2 is expressed in CNS neurons [8, 18, 19]. There is some evidence for VMAT1 expression in the hippocampus and subcortical structures in the brain. These data suggest that VMAT1 plays a key role in survival of hippocampal neurons and might contribute to neurocognitive deficits observed in neuropsychiatric disorders [34]. It is widely accepted that VMAT2 is the main vesicular transporter involved in packaging DA into vesicles [8, 18, 19].

The dopamine cycle balance can be disturbed by ischemic injury, which causes a significant reduction in VMAT and DAT. NBP reverses this reduction, even returns these two proteins to normal levels. Although the high expression of DAT might reduce the dopamine in synaptic cleft, the recovery of VMAT function by NBP increases dopamine release and rebuilds dopamine cycle and balance at a high level, which contributes to keeping dopamine at a stable level and protects the neurons' function and activity.

It should be noticed that, given the time required for transcription, translation, and membrane trafficking and axonal transport of these transporters, it is difficult to attribute the early dopamine increase to increased transporter abundance. The early surge might be attributed to rapid functional regulation, such as transporter phosphorylation status, altered neuronal firing, changes in dopamine metabolism, or indirect effects of NBP on improved tissue viability (including superior ROS scavenging, mitochondria protection, neuroinflammation inhibition and neuronal apoptosis).

Glutamate cytotoxicity is one of the main contributing factors in cerebral ischemic injury. Excessive release of the neurotransmitter glutamate induces excitotoxicity, which in turn leads to Ca^2+^ overload, oxidative stress, mitochondrial dysfunctions and eventually neuronal death [35].

The balance of major excitatory and inhibitory is named excitatory and inhibitory (E/I), which mainly mediated by glutamate and γ‐aminobutyric acid (GABA) [36, 37, 38]. The mEPSC/mIPSC frequency is often used as an indicator of E/I balance [22]. Excessive release of glutamate disturbs this balance during cerebral ischemic injury and might contribute to the excitotoxic injury cascade. According to our data, NBP can inhibit the excessive release of glutamate and recover the balance. Both D_1_R and adenosine A_1_Rs might contribute to the restoration of synaptic function.

Dopamine receptors can be divided into two main groups—D_1_‐like receptor (D_1_R, D_5_R) and D_2_‐like receptor (D_2_R, D_3_R, D_4_R). The activation of D_1_R by systemic administration confers neuroprotection [29]. D_2_R activation also has been defined as a neuroprotective strategy for cerebral injury [30]. These receptors can be activated by dopamine. Interestingly, the activity of D_2_R and its downstream pathway is more easily inhibited by high levels of dopamine than D_1_R [39]. So during the acute cerebral ischemic injury, increasing dopamine might mainly activate D_1_R.

The activation of D_1_R depresses excitatory synaptic dysfunction induced by ischemia through activation of PKA and adenosine A_1_R. Adenosine is an endogenous purine nucleoside. Its receptors including A_1_R, A_2_AR, A_2_BR, and A_3_R [40]. In the brain, the activation of A_1_Rs reduces Ca^2+^ influx, then inhibits the presynaptic function, reduces the neuronal excitability and excitatory neurotransmitters release [40, 41]. A_1_Rs activation has neuroprotection against glutamate‐induced cytotoxicity and related cerebral ischemia. On the other side, our data showed that A_1_Rs inhibition aggravated cerebral ischemic injury. This inhibition also eliminated the restoration of synaptic function and almost abolished the neuroprotection induced by NBP.

## 5 Conclusion

The neuroprotective effect of NBP against cerebral ischemic injury may involve, at least in part, the upregulation of the VMAT‐DAT‐dopamine cycle, which appears to contribute to the amelioration of synaptic dysfunction. The activation of D_1_R and adenosine A_1_Rs seems to be engaged in the downstream signaling cascades.

## Author Contributions

Ai‐Jun Liu and Ding‐Feng Su conceived the idea; Ai‐Jun Liu and Yi‐Ling Su designed the experiments; Xiao‐Ting Zhou, Qin Liu and Ruo‐Xi Zhang performed the experiments including animal experiments, immunoblotting and the detection of neurotransmitters; Ming Chen performed whole‐Cell Voltage‐Clamp recording. Qin Liu and Xiao‐Ting Zhou analyzed the data; Ai‐Jun Liu wrote the paper.

## Funding

This work was supported by National Natural Science Foundation of China, 82273984. National Key Research and Development Program of China, 2019YFC1708504. Three Year Action Plan for Shanghai to Further Accelerate the Inheritance, Innovation and Development of Traditional Chinese Medicine (2025–2027), (No. 1‐1‐2).

## Ethics Statement

All animal experiments were conducted in accordance with the guidelines approved by the Yueyang Hospital Animal Care Committee, Shanghai University of Traditional Chinese Medicine (YYLAC‐2023‐203).

## Conflicts of Interest

The authors declare no conflicts of interest.

## Supporting information


**Figure S1:** NBP reduced the infarct size in mice with MCAO. 1% TTC staining images of coronal brain sections from MCAO mice. Animals were randomly divided into 4 groups, vehicle control and NBP (40, 80 and 160 mg/kg, *n* = 10). Three animals were discarded because of death (control) or low CBF reduction (40 and 160 mg/kg group).
**Figure S2:** NBP did not affect the loss of TH immunostaining positive cells in MCAO mice (A) Representative photographs of TH immunostaining in the substantia nigra and ventral tegmental area (VTA). (B) TH immunostaining in hippocampus. (C) Quantitative comparison of TH immunostaining positive cells in substantia nigra pars compacta (SNpc). Scale bar: the upper part of A, 500 μm, the lower part of A, 100 μm; B, 200 μm, n = 5. Data are shown as mean ± SD and analyzed by one‐way ANOVA, followed by Dunnett's test. *p < 0.05 versus sham operation.
**Figure S3:** Representative photographs of TH immunostaining in the hippocampus. (A) sham, (B) model, (C) NBP (80 mg/kg). Scale bar: 50 μm, n = 5.
**Figure S4:** The reduction of glutamate by NBP was abolished by PSB36 in MCAO mice. Microdialysis detection in the ischemic penumbral area of hippocampus shows the extracellular levels of glutamate in 4 groups (n = 5 in each group). Data are shown as mean ± SD and analyzed by one‐way ANOVA, followed by Tukey's post hoc test. **p < 0.05 versus vehicle control, ns, no significance.

## Data Availability

The data that support the findings of this study are available on request from the corresponding author. The data are not publicly available due to privacy or ethical restrictions.
