# Supplementary material for: VMAT‐DAT‐Dopamine Regulatory System Involved in the Protective Effect of 3‐n‐Butylphthalide Against Ischemic Stroke
Source: CNS Neurosci Ther. 2026 Aug 18;32(8):e71094. doi: 10.1002/cns.71094 (PMC13486960; doi:10.1002/cns.71094)
Supplement: Supplementary file 1 [file CNS-32-e71094-s001.docx]

**Supplementary Figures**

**Supplementary Figure 1.** **NBP reduced the infarct size in mice with MCAO.** 1% TTC staining images of coronal brain sections from MCAO mice. Animals were randomly divided into 4 groups, vehicle control and NBP (40, 80 and 160 mg/kg, n=10). Three animals were discarded because of death (control) or low CBF reduction (40 and 160 mg/kg group).

**Supplementary Figure 2. NBP did not affect the loss of TH immunostaining positive cells in MCAO mice**

**A.** Representative photographs of TH immunostaining in the substantia nigra and ventral tegmental area (VTA). B. TH immunostaining in hippocampus. C. Quantitative comparison of TH immunostaining positive cells in substantia nigra pars compacta (SNpc). Scale bar: the upper part of A, 500 μm, the lower part of A, 100 μm; B, 200 μm, n=5. Data are shown as mean ± SD and analyzed by one-way ANOVA, followed by Dunnett’s test. **P* < 0.05 v.s. sham operation.

**Supplementary Figure 3.** Representative photographs of TH immunostaining in the hippocampus. A. sham, B, model, C, NBP (80 mg/kg). Scale bar: 50 μm, n=5.

**Supplementary Figure 4. The reduction of glutamate by NBP was abolished by PSB36 in MCAO mice.**

Microdialysis detection in the ischemic penumbral area of hippocampus shows the extracellular levels of glutamate in 4 groups (n=5 in each group). Data are shown as mean ± SD and analyzed by one-way ANOVA, followed by Tukey’s post-hoc test. ***P* < 0.05 v.s. vehicle control, n.s., no significance.
